# Towards Development of Clustering Applications for Large-Scale Comparative Genotyping and Kinship Analysis Using Y-Short Tandem Repeats

**DOI:** 10.1089/omi.2014.0136

**Published:** 2015-06-01

**Authors:** Ali Seman, Azizian Mohd Sapawi, Mohd Zaki Salleh

**Affiliations:** ^1^ Integrative Pharmacogenomics Institute (iPROMISE), Faculty of Computer and Mathematical Sciences Universiti Teknologi MARA (UiTM), Shah Alam, Selangor, Malaysia.; ^2^ Center for Computer Science Studies, Faculty of Computer and Mathematical Sciences Universiti Teknologi MARA (UiTM), Shah Alam, Selangor, Malaysia.

## Abstract

Y-chromosome short tandem repeats (Y-STRs) are genetic markers with practical applications in human identification. However, where mass identification is required (e.g., in the aftermath of disasters with significant fatalities), the efficiency of the process could be improved with new statistical approaches. Clustering applications are relatively new tools for large-scale comparative genotyping, and the *k*-Approximate Modal Haplotype (*k*-AMH), an efficient algorithm for clustering large-scale Y-STR data, represents a promising method for developing these tools. In this study we improved the *k*-AMH and produced three new algorithms: the N*k*-AMH I (including a new initial cluster center selection), the N*k*-AMH II (including a new dominant weighting value), and the N*k*-AMH III (combining I and II). The N*k*-AMH III was the superior algorithm, with mean clustering accuracy that increased in four out of six datasets and remained at 100% in the other two. Additionally, the N*k*-AMH III achieved a 2% higher overall mean clustering accuracy score than the *k*-AMH, as well as optimal accuracy for all datasets (0.84–1.00). With inclusion of the two new methods, the N*k*-AMH III produced an optimal solution for clustering Y-STR data; thus, the algorithm has potential for further development towards fully automatic clustering of any large-scale genotypic data.

## Introduction

Y-chromosome short tandem repeats (Y-STRs) are a class of genetic markers found only on the male-specific Y chromosome. Y-STR data have many practical applications, in particular for distinguishing lineages and providing information about lineage relationships (Kayser et al., 2004), and for genetic genealogy in general (Perego, 2005). Commercial companies such as Family Tree DNA (www.familytreedna.com) and Genebase (http://www.genebase.com) have taken advantage of these data by offering Y-STR genealogical testing kits as a means of providing genetic proof in genealogy.

In forensic genetics, Y-STRs are of primary concern in cases where human identification is necessary, for example, in rape and sexual assault cases (Betz et al., 2001), paternity testing (Rolf et al., 2001), missing person cases (Dettlaff-Kakol and Pawlowski, 2002), identification of human migration patterns (Stix, 2008), and reexamination of ancient cases (Gerstenberger et al., 1999). However, in cases that require a process of mass identification, such as in the aftermath of tsunamis or aviation accidents, the efficiency of the identification process could be improved with new approaches such as data mining (Leclair, 2004).

Most applications that employ Y-STR data are based primarily on a direct comparison by using pair-wise analysis, which is easily achieved if the sample size is relatively small. However, where the sample size is large and/or there are multiple samples, multi-criteria analyses such as supervised and unsupervised learning methods produce results that are more informative. Indeed, several methods for grouping multiple samples of Y-STR data automatically have been reported (Schlecht et al., 2008; Seman et al., 2010a; 2012; 2013a). In the supervised learning method, Y-STR data can be classified by haplogroup via the decision tree method (Schlecht et al., 2008; Seman et al., 2013a), Bayesian modeling, and support vector machines (Schlecht et al., 2008). Similarly, unsupervised learning methods can be used to cluster Y-STR data by similar genetic distances (Seman et al., 2010a; 2010b; 2010c; 2010d; 2012).

In a detailed comparison (Seman et al., 2012), a recently developed clustering algorithm, the *k*-Approximate Modal Haplotype (*k*-AMH) algorithm, produced optimal results compared to eight other clustering algorithms including the *k*-Modes (Huang, 1998), fuzzy *k*-Modes (Huang and Ng, 1999), and new fuzzy *k*-Modes (Ng and Jing, 2009). The optimization procedure for the *k*-AMH algorithm, which uses the data and not the mode mechanism as the center of clusters, significantly improves the overall clustering results. Indeed, the advantages of the *k*-AMH algorithm are the use of objects (the data) as the center (medoid) instead of modes, as well as the new cost function and procedure used to determine the final center of clusters. This procedure is in contrast to the mode mechanism used in the common *k*-Modes-type algorithms listed above.

In this study we aimed to improve the *k*-AMH algorithm by introducing two new methods: a method for initial selection of the cluster center and a dominant weighting method. The first method replaces the randomized method in the *k*-AMH algorithm; the second method replaces the original dominant weighting method. Consequently, the improved algorithm was proposed for use in the further development of clustering applications for large-scale comparative genotyping and kinship analysis using Y-STR.

In the following sections of this article, we first describe the fundamental features of the *k*-AMH algorithm and the two methods proposed above, as well as the Y-STR data and clustering evaluation methods. Subsequently, we present the clustering results by comparing the performance of (a) the original *k*-AMH algorithm; (b) the *k*-AMH algorithm with the new method for the initial selection of the cluster center, which we call N*k*-AMH I; (c) the *k*-AMH algorithm with the new dominant weighting method, named N*k*-AMH II; and (d) the *k*-AMH algorithm combining these two new methods, known as N*k*-AMH III. Finally, we discuss the potential applications to be further developed for the fully automatic clustering of large-scale genotypic data.

## Materials and Methods

### k-AMH algorithm

Consider $X = \{ X_1 , X_2 , \ldots , X_n \}$ to be a set of *n* Y-STR objects and $H = \{ H_1 , H_2 , \ldots , H_k \}  \in X$ to be a set of approximate modal haplotypes. The goal of the *k*-AMH algorithm is to find *k* clusters in *n* Y-STR objects by first randomly selecting an object to be the medoid, *h*, for each cluster. Next, the objects, *X*, are iteratively replaced one-by-one towards *h*. The replacement is based on the maximum cost function as simplified by *P(W,D)* and described in Equation 1 and is maximized subject to Equations (2), (3), (4), (4a), and (4b).

\begin{align*} P ( W , D ) ^r > P ( W , D ) ^t , r \neq t ; \forall t
, \it 1 \le t \le ( n - k ) \tag{{\rm Eq.} \ 1} \end{align*}

The $$ P ( W , D )$$ is a cost function as described in Equation 2

\begin{align*} P ( W , D ) = \mathop \sum \limits_{l = 1}^k \mathop
\sum \limits_{i = 1}^n w_{li}^{ \alpha} d_{li} \tag{{\rm Eq.} \ 2} \end{align*}

where:

• $w_{li}^{ \alpha} \in W$ a is a (*k*×*n*) fuzzy membership matrix that denotes the degree of membership of object *i* in the *l^th^* cluster, which contains a value of 0 to 1 as described in Equation 3.

\begin{align*} w_ { li } ^ { \alpha } = \begin{cases} 1 \quad \quad
\quad \quad X_i = H_l \\ 0 \quad \quad \quad \quad X_i = H_z , z \neq l \\ { \left[ \mathop\sum \limits_ { z = 1 } ^k \frac { d ( X_i , H_i )}  { d ( X_i , H_z ) } \right] ^ \frac { - \alpha }  { \alpha - 1 } } \quad Otherwise \end{cases} \tag {{\rm Eq. } \ 3 } \end{align*}

where *k* (*≤n*) is a known number of clusters, *H* is *th*e medoid, $$ \alpha \in \ [ 1 , \infty )$$ is a weighting exponent. [Note that this alpha is typically based on 1.1, 1.2, 1.3, 1.4, 1.5, 1.6, 1.7, 1.8, 1.9, and 2.0, as introduced by Huang and Ng (1999)], and $$ d ( X_i , H_z )$$ is the distance measured between the object *X_i_* and the medoid *H_z_*.

• $d_{li} \in D$ is another (*k×n*) partition matrix with a dominant weighting value of 1.0 or 0.5. The dominant weighting value, *d_li_* is described in Equation 4, subject to Equations 4a and 4b.
  \begin{align*} d_{li} = \begin{cases} 1.0 , \quad if \ w_{li}^{
\alpha} = \max^{w_{li}^{\alpha} , {1 \le l \le k}} \\ 0.5 , \quad
otherwise \end{cases} \tag{{\rm Eq.}\ 4}\end{align*}

subject to:

\begin{align*} 1.5 \le \mathop \sum \limits_{l = 1}^k d_{li} \le
k , 1 \le i \le n \tag{{\rm Eq.} \ 4{\rm a}}\end{align*}

\begin{align*} 0.5 < \mathop \sum \limits_{i = 1}^n d_{li} < n ,
1 \le l \le k \tag{{\rm Eq.}\ 4{\rm b}}\end{align*}

The optimization procedure for the *k*-AMH algorithm is shown as follows.

Step 1: Choose randomly an initial center selection called the approximate modal haplotype, $$ H^{ ( 1 ) } \in X.$$ Calculate $$ P ( W , D )$$. Set *t=1*.
Step 2: Choose *X*^(*t*+1)^ such that *P*(*W, D*)^*t*+1^ is maximized. Replace $H^{ ( 1 ) } \leftarrow X^{ ( t + 1 ) }$.
Step 3: Set *t=t+1*. Stop when *t=n (the number of objects)*; otherwise go to Step 2.

A detailed description of the *k*-AMH algorithm can be found in Seman et al., (2012).

### The new initial center selection method

In unsupervised algorithms such as *k*-Mean- and *k*-Mode-type algorithms, the randomized initial center selection is one of the factors contributing to the quality of clustering results (Li et al., 2008). However, in a previous study, using the *k*-AMH algorithm with a randomized initial center, selection produced accuracy scores with higher minimum values compared with those of all other tested clustering algorithms (Seman et al., 2012). Because of the nature of Y-STR data, which are dominated by a high degree of similarity, the probability of obtaining initial center selections that are close together is relatively high. Under these circumstances, the *k*-AMH algorithm is apparently not sensitive to initial selection centers, despite their being identical or similar to each other. Therefore, the aim of the new initial center selection method proposed here was to select any identical or similar initial center selections. The detailed steps used to select these initial center selections are described in Equations 5, 5a, 6, and 6a.

Step 1: Find a mode, *M*, from the data, *X*, such that

\begin{align*} m_{1 , j} = a_j^{ ( r ) } \tag{{\rm Eq.} \ 5} \end{align*}

where $$ m_{1 , j} \in M$$ and $$ a_j^{ ( r ) }$$ is the mode of attribute values of *A_j_* in object *X_i_*, such that

\begin{align*} f \left( a_j^{ ( r ) } \mid X_i \right) \ge f \left(a_j^{ ( t ) } \mid X_i \right) \forall t , 1 \le t \le p_j , a_j^{ ( r ) } \neq a_j^{ ( t ) } , \tag{{\rm Eq.} \ 5{\rm a}}\end{align*}

where *p* is the number of categories of attribute *A_j_* for *1≤j≤m*

Step 2: Calculate the distance between *X* and *M*, such that

\begin{align*} dist ( M , X ) = \mathop \sum \limits_{i = 1}^n \delta
( m_1 , X_i ) \tag{{\rm Eq.} \ 6} \end{align*}

where

\begin{align*} \delta ( m_{1 , j} , x_{i , j} ) = \begin{cases}0, \quad m_{i, j} = x_{i , j} \\ 1, \quad
m_{i, \,j} \neq x_{i , j}\end{cases}  \tag{{\rm Eq.} \ 6{\rm
a}}\end{align*}

where *1≤i≤n* and *1≤j≤m*

Step 3: Choose the initial center clusters, *H*, with the top-*k* smallest distances.

### The new dominant weighting method

The dominant weighting method of the *k*-AMH algorithm described in Equation 4 is a simple weighting value with either a 50% or 100% probability that an object belongs to a cluster. In fuzzy sense, the maximum membership value (any object that is closer to its cluster) is very subjective. Where membership values fall between 1/*k* to 1.0 (*k* is the number of clusters), the probability cannot be 100%. The only condition in which the object belongs to its cluster with 100% confidence is when the maximum membership value is 1.0 (i.e., the object is identical to its cluster center). This is the drawback of the dominant weighting value imposed by the *k*-AMH algorithm. Therefore, in order to improve the original dominant weighting value, we considered the cut-off point of the upper quartile (Q3) or 75^th^ percentile to be given to any maximum values less than 1.0, with a condition that the values must be greater than 1/k, where k is the number of clusters; otherwise, a cut-off point of median or 50^th^ percentile is given. The new dominant weighting value method is described in Equation 7.

\begin{align*} d_{li} = \begin{cases} 1.00 \quad if \ w_{li}^
\alpha = {\it max}^{w_{li}^\alpha 1 \le l \le k} = 1.00 \\ 0.75
\quad if \ w_{li}^\alpha = {\it max}^{w_{li}^\alpha 1 \le l \le k}
> 1/k \\ 0.50 \quad otherwise \end{cases} \tag{{\rm Eq.}\
7}\end{align*}

### Y-STR datasets

For benchmarking results, six Y-STR datasets were used to compare the clustering performances of all algorithms. These datasets were divided into two categories: (1) Y-STR datasets for haplogroup applications (datasets 1–3; Table 1), and (2) Y-STR dataset for Y-surname applications (datasets 4–6; Table 1). The main difference between these two categories was their similarity distance; the Y-STR haplogroup data were typically more distinct than the Y-STR surname data, having higher similarity distances [for a detailed description and explanation of the characteristics of the data, see Seman et al., (2013)].

**Table 1.** Summary of Y-STR Datasets

*Dataset*	*Number of objects*	*Number of classes*	*Distribution of objects*
1	751	5	E (24), G (20), L (200), J (32), and R (475)
2	267	4	L (92), J (6), N (141), and R (28)
3	263	3	G (37), Group N (68), and Group T (158)
4	236	4	D (112), F (64), M (42), and W (18)
5	112	8	G2 (30), G4 (8), G5 (10), G8 (18), G10 (17), G16 (10), G17 (12), and G29 (7)
6	112	14	G2 (9), G10 (17), G15 (6), G18 (6), G20 (7), G23 (8), G26 (8), G28 (8), G34 (7), G44 (6), G35 (7), G46 (7), G49 (10), and G91 (6)

### Evaluation method

The analyses are based on the mean accuracy scores obtained from experiments that were run 100 times (100-run) for each algorithm and dataset. During the 100-run experiments, the Y-STR objects in the datasets were randomly reordered from the original order of each run. The misclassification matrix was used to analyze the correspondence between the clusters and the classes of the instances. For example, if a particular dataset had four classes (groups) such as A, B, C, and D, then the objective was to cluster the dataset into four clusters. If the number of objects belonging to the classes A, B, C, and D was 10, 10, 10, and 17, respectively, a 100% accuracy score would be achieved if all objects were in their respective clusters, as shown in Table 2.

**Table 2.** Misclassification Matrix that Produces a 100% Clustering Accuracy Score

	*Cluster 1*	*Cluster 2*	*Cluster 3*	*Cluster 4*	*Total*
A	0	0	**10**	0	10
B	0	0	0	**10**	10
C	**10**	0	0	0	10
D	0	**17**	0	0	17
Total	10	17	10	10	47

Thus, the performance of the algorithms was primarily measured by the clustering accuracy, defined by Huang (1998) as described in Equation 8.

\begin{align*}Ac = \frac { \mathop \sum \limits_ { l = 1 } ^k a_l }  { n } \tag { \rm Eq.\ 8 } \end{align*}

where *k* is the number of clusters, *a_l_* is the number of instances occurring in cluster *l* and its corresponding group (haplogroup or surname), and *n* is the number of instances in the datasets.

In addition to the analysis described above, we performed secondary analysis using precision and recall methods. These methods are described in Equations 9 and 10, respectively.

\begin{align*}Pr = \frac { \mathop \sum \limits_ { l = 1 } ^k \left( \frac { a_l }  { a_l + b_l } \right) }  { n } \tag { { \rm Eq. } \ 9 } \end{align*}

\begin{align*}Rc = \frac { \mathop \sum \limits_ { l = 1 } ^k \left( \frac { a_l }  { a_l + c_l } \right) }  { n } \tag { { \rm Eq. } \ 10 } \end{align*}

where b_i_ is the number of incorrectly classified objects, and c_i_ is the number of objects in a given class but not in a cluster.

## Results

Here, we present results based on accuracy, precision, and recall scores taken from 100 experimental runs for each dataset and algorithm. Table 3 shows the clustering accuracy results for the six datasets. The results showed that, in general, combining the two new methods in the N*k*-AMH III algorithm significantly increased the mean accuracy scores for Dataset 1 (1% increase), 2 (5%), 3 (1%), and 6 (3%), while Datasets 4 and 5 maintained their optimum accuracy score of 1.0. By combining all six datasets, the N*k*-AMH III achieved a mean accuracy score of 0.95, representing an increase of 2% compared with the original *k*-AMH algorithm (0.93).

**Table 3.** Clustering Accuracy Scores for All Datasets^*^

		*Dataset*						
*Accuracy*	*Algorithm*	*1*	*2*	*3*	*4*	*5*	*6*	*Combined mean*
Mean	*k*-AMH	0.83	0.93	0.96	**1.00**	**1.00**	0.87	0.93
	N*k*-AMH I	0.82	0.93	**0.97**	**1.00**	**1.00**	0.87	0.93
	N*k*-AMH II	0.82	0.94	0.95	**1.00**	**1.00**	**0.91**	0.94
	**N*k*-AMH III**	**0.84**	**0.98**	**0.97**	**1.00**	**1.00**	0.90	**0.95**
Min.	*k*-AMH	0.80	0.92	0.95	**1.00**	**1.00**	0.79	0.91
	N*k*-AMH I	**0.81**	0.93	**0.97**	**1.00**	**1.00**	0.82	0.92
	N*k*-AMH II	0.78	0.75	0.94	**1.00**	**1.00**	0.86	0.89
	**N*k*-AMH III**	**0.81**	**0.98**	0.96	**1.00**	**1.00**	**0.88**	**0.94**
Max.	*k*-AMH	**0.85**	**0.99**	**0.97**	**1.00**	**1.00**	0.91	**0.95**
	N*k*-AMH I	**0.85**	0.93	**0.97**	**1.00**	**1.00**	0.88	0.94
	N*k*-AMH II	**0.85**	0.98	**0.97**	**1.00**	**1.00**	**0.92**	**0.95**
	**N*k*-AMH III**	**0.85**	0.98	**0.97**	**1.00**	**1.00**	**0.92**	**0.95**

^*^Mean, min., and max. represent the mean, minimum, and maximum accuracy scores taken from 100 experimental runs for each dataset.

Considering the implementation of the new methods in the original *k*-AMH algorithm (i.e., result from N*k*-AMH I and N*k*-AMH II), both algorithms showed inconsistent performance. The N*k*-AMH I significantly increased the minimum values of the accuracy scores for each dataset when compared with the *k*-AMH algorithm. These results indicate that the new initial center selection method was a main factor contributing to the overall performance of the N*k*-AMH I algorithm. In contrast to the minimum accuracy scores, N*k*-AMH I produced maximum values for accuracy scores that were slightly decreased compared with *k*-AMH. For N*k*-AMH II, the mean clustering accuracy score when the six datasets were combined was 0.94, a 1% increase on the *k*-AMH algorithm (0.93). However, the minimum values of the accuracy scores produced by N*k*-AMH II decreased slightly compared with *k*-AMH, whereas the two algorithms produced similar maximum values for accuracy scores.

Table 4 shows an additional comparison based on precision and recall scores for each dataset and algorithm. N*k*-AMH III produced a mean precision score of 0.93 when the six datasets were combined, which was a significant increase of 5% compared with *k*-AMH, 7% compared with N*k*-AMH I, and 4% compared with N*k*-AMH II. Furthermore, N*k*-AMH III produced a recall score of 0.93 for the six datasets combined, which was a significant increase of 7% compared with *k*-AMH, 5% compared with N*k*-AMH I, and 2% compared with N*k*-AMH II.

**Table 4.** Clustering Precision and Recall Scores for Individual Datasets and Combined Datasets

		*Dataset*						
	*Algorithm*	*1*	*2*	*3*	*4*	*5*	*6*	*Combined mean*
Mean (Precision)	*k*-AMH	0.67	0.75	**0.97**	**1.00**	**1.00**	0.87	0.88
	N*k*-AMH I	0.72	0.66	0.95	**1.00**	**1.00**	0.85	0.86
	N*k*-AMH II	0.68	0.86	0.92	**1.00**	**1.00**	**0.90**	0.89
	**N*k*-AMH III**	**0.81**	**0.93**	0.95	**1.00**	**1.00**	0.89	**0.93**
Mean (Recall)	*k-*AMH	0.68	0.68	0.94	**1.00**	**1.00**	0.85	0.86
	N*k-*AMH I	0.69	0.75	**0.98**	**1.00**	**1.00**	0.85	0.88
	N*k-*AMH II	0.70	0.88	0.96	**1.00**	**1.00**	0.93	0.91
	**N*k-*AMH III**	**0.73**	**0.94**	0.97	**1.00**	**1.00**	**0.92**	**0.93**

These results indicate that the two newly proposed methods should not be implemented alone. Indeed, the combination of these methods in N*k*-AMH III produced the most reliable algorithm for clustering Y-STR data. To test this result further, we performed a one-way ANOVA. In this test, we found that the assumption of homogeneity of variance was violated (Levene *F*(3, 2396)=7.41, *p*<0.01); therefore, we reported the Welch *F*-ratio. There was significant variance in the clustering accuracy scores among the four algorithms (*F*(3, 1328)=10.39, *p*<0.01); therefore, we used the Games–Howell procedure for a multiple comparison. Table 5 shows the results of this comparison, comparing the N*k*-AMH III algorithm against the others. Performance of N*k*-AMH III (*M*=0.95, 99% CI [0.94, 0.95]) differed significantly from the other three algorithms (*p*<0.01); thus, it performed significantly better than the other algorithms when clustering Y-STR data.

**Table 5.** One-Way ANOVA Comparison of N*k*-AMH III with Three Other *k*-AMH Algorithms (*k*-AMH, N*k*-AMH I, and N*k*-AMH II)

*(I) Algorithm*	*(J) Algorithm*	*Mean difference (I-J)*	*Std. error*	P *value*
N*k*-AMH III	1. *k*-AMH	0.02	0.0036	<0.0001
	2. N*k*-AMH I	0.02	0.0036	<0.0001
	3. N*k*-AMH II	0.01	0.0037	0.0060

**FIG. 1.**
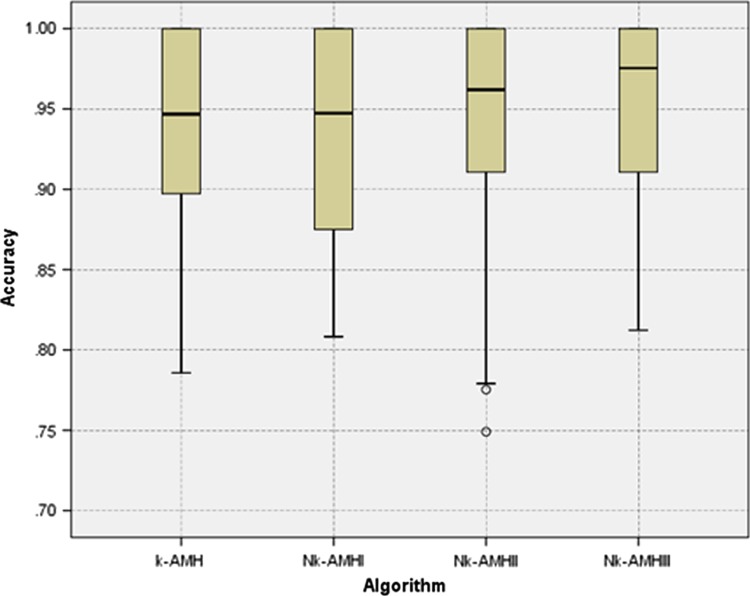
Box plot comparison of the clustering accuracy performance of k-AMH, Nk-AMH I, Nk-AMH II, and Nk-AMH III algorithms.

Figure 1 shows a direct performance comparison for all *k*-AMH-type algorithms. The range of the box plot for the N*k*-AMH III algorithm was relatively small, which represents greater consistency, and its median and minimum accuracy scores were higher than the other *k*-AMH algorithms. We suggest that the superior performance of N*k*-AMH III algorithm was essentially caused by the combination of our two new methods. These new methods are the main factors in the N*k*-AMH III algorithm, which can handle the uniqueness of Y-STR data characterized by objects with high similarity.

## Discussion

Owing to its superior performance in clustering Y-STR data, the N*k*-AMH III algorithm incorporating unsupervised methods has the potential to be the core engine moving towards the development of fully automatic clustering applications for large-scale genotypic data. Furthermore, it could also be applied to any genotypic data with categorical attributes such as autosomal STR, SNP, and DNA sequences.

In this study, N*k*-AMH III produced high accuracy scores and showed consistency in the results, suggesting that the algorithm can be considered for the future development of several clustering applications. For example, the algorithm could be applied to develop a clustering tool for forensic identification in the analysis of mass fatality incidents, which would be particularly useful in cases where identification must be achieved solely by using DNA evidence. With N*k*-AMH III, the tool could potentially group any set of genotypic data (e.g., STR, SNP) in such a way that data from the same group are more similar to each other than to those from other groups. When dealing with large-scale comparative genotyping and kinship analysis, such a tool could quickly separate genotypic data into groups according to the similarities in the data. Therefore, the N*k*-AMH III algorithm could be applied as a complementary tool with the existing tools of mass identification such as MDKAP, MFISys, and DNAVIEW, particularly for kinship data analyses.

These three established applications have their own advantages in mass identifications (e.g., MDKAP is good for kinship analysis through pair-wise comparison, MFISys has an advantage of collapsing and sorting datasets, and DNA VIEW is able to determine kinship by pedigree analysis). In addition, the N*k*-AMH III application has also several advantages. For example, it would be useful for providing a quick overview of intra-group and inter-group similarities, which could be used during the pre-processing stages of mass identification. Moreover, the approximate modal haplotypes obtained by the algorithm as the center of clusters could also be used as reference samples for those that belong to the clusters, especially in cases where the DNA samples are collected from unknown remains. Furthermore, the predefined number of clusters, ranging from two to any given maximum, could assist researchers to better identify closeness.

Another application for the N*k*-AMH III algorithm could be in the development of a tool for clustering large haplogroups (i.e., groups of similar haplotypes that share a common ancestor). Anthropologists could use a preliminary tool such as this to automate clustering of a group of similar patterned and related descendant haplotypes that share a common ancestor. Indeed, the approximate modal haplotypes obtained by using the algorithm would be a good reference as candidate modal haplotypes for particular haplogroups. In addition to clustering haplogroups, the algorithm could also be used to develop a tool for identifying family trees. Within its capabilities, any genotypic data could be submitted to any family database for the algorithm to automatically identify groups of similar family relatedness.

## Conclusions

Of the algorithms tested in this study, the N*k*-AMH III algorithm proved to be the superior unsupervised k-AMH algorithm for clustering Y-STR data. The algorithm significantly increased the minimum values of accuracy scores, as well as the overall accuracy scores. Moreover, in general, the algorithm significantly increased mean clustering accuracy scores. The superior performance of the N*k*-AMH III can be explained by the newly introduced initial center selection, and dominant weighting value. In addition, the use of objects as the center of clusters in the *k*-AMH procedure is an important factor that contributes to its optimal performance when processing unique Y-STR data with high similarity distances. With an error rate of just 5% when clustering Y-STR data, the use of the N*k*-AMH III algorithm in future research for further development of clustering tools applicable to forensic human identification is conceptually feasible, especially for the analysis of mass fatality incidents. If these applications are to be developed, researchers will need to consider integrating other methods (e.g., for statistical analyses and/or reporting of results) with the algorithm. Nevertheless, implementing the algorithm within the aforementioned clustering tools is experimentally feasible.
